# Sialylation-Siglec glyco-immune checkpoints in hepatocellular carcinoma: mechanisms of immune evasion, therapeutic resistance, and postoperative recurrence

**DOI:** 10.3389/fimmu.2026.1967178

**Published:** 2026-10-28

**Authors:** Kai Zhang, Longhao Zhang, Nani Long, Haitao Zhao, Xin Lu, Yiyao Xu

**Affiliations:** 1 Department of Liver Surgery, Peking Union Medical College Hospital, Chinese Academy of Medical Sciences & Peking Union Medical College (CAMS & PUMC), Beijing, China; 2 Department of Cardiothoracic Surgery Intensive Care Unit, The First Affiliated Hospital of Guangxi Medical University, Nanning, Guangxi, China

**Keywords:** hepatocellular carcinoma, immune escape, natural killer cells, sialylation, Siglec, tumor-associated macrophages

## Abstract

Postoperative recurrence and incomplete responses to immunotherapy remain major obstacles to the long-term control of hepatocellular carcinoma (HCC). Tumor-associated sialylation may promote immune escape by generating glycans that bind inhibitory members of the sialic acid-binding immunoglobulin-like lectin (Siglec) family on immune cells. This review critically examines HCC-specific evidence on abnormal sialylation, Siglec-related dysfunction of natural killer cells and macrophages, treatment-associated glycan remodeling, and possible links to molecular residual disease and postoperative recurrence. Glycan patterns vary substantially between tumors and within individual tumors. Total sialic acid abundance and the expression of a single sialyltransferase therefore provide only part of the information needed to identify functional Siglec ligands. Their activity also depends on glycan structure, chemical modifications, the molecule carrying the glycan, ligand density, surface accessibility, and spatial organization. The strongest HCC-specific evidence concerns the interaction between Siglec-9 and natural killer cells. Supporting findings include receptor and ligand expression, close spatial proximity within tumors, associations with adverse clinical outcomes, and recovery of natural killer-cell activity after pathway inhibition in preclinical models. Siglec-10-high tumor-associated macrophages are associated with an immunosuppressive myeloid state. Their endogenous ligands and receptor-specific roles *in vivo* require further characterization. In sorafenib-resistant HCC models, increased ST3 β-galactoside α-2,3-sialyltransferase 1 (ST3GAL1) expression accompanies greater Siglec-7 and Siglec-9 probe binding, while ST3GAL1 silencing improves natural killer-cell-mediated cytotoxicity. These findings support a functional contribution of altered glycosylation to treatment-related immune escape. Glycocalyx-dependent changes in adhesion, migration, and survival may also contribute to dissemination and residual-cell persistence alongside immune evasion. Direct studies are now needed to determine whether sialylation-Siglec signaling helps residual HCC cells survive after curative-intent therapy. Clinical translation will require identification of specific glycan-carrier pairs, spatial mapping of receptor-ligand contacts, receptor-specific validation in species-appropriate models, and prospective evaluation of glyco-immune biomarkers alongside circulating tumor DNA and established clinicopathological factors across HCC etiologies. Overall, current evidence identifies the sialylation-Siglec system as a biologically plausible and cell-specific immune-evasion network. It also provides a framework for biomarker-guided evaluation of Siglec-directed therapies and tumor-targeted desialylation and future studies of its contribution to HCC recurrence.

## 1 Introduction

Hepatocellular carcinoma (HCC) accounts for the majority of primary liver cancers and remains a major cause of cancer-related mortality worldwide (1, 2). For selected patients with early-stage HCC, liver resection, liver transplantation, and local ablation are potentially curative treatments that can provide long-term disease control (2). Nevertheless, recurrence remains common and limits durable survival (3, 4). Clinicopathological studies have identified two broad patterns of recurrence. Early recurrence is commonly associated with microvascular invasion (MVI), satellite lesions, aggressive tumor features, and occult intrahepatic dissemination, whereas late recurrence is more consistent with *de novo* hepatocarcinogenesis in the chronically diseased liver (3, 4). These patterns indicate that HCC recurrence has more than one biological basis. In particular, early recurrence suggests that residual or disseminated tumor cells can persist within the hepatic microenvironment. Therefore, long-term postoperative control may depend not only on the extent of tumor removal but also on whether hepatic immune surveillance can eliminate residual or disseminated tumor cells (3, 4).

Evidence from advanced HCC provides a separate but complementary perspective on tumor persistence. Immune checkpoint inhibitors targeting programmed cell death protein 1 (PD-1), programmed death-ligand 1 (PD-L1), or cytotoxic T-lymphocyte-associated protein 4 (CTLA-4) have become an important component of systemic treatment for advanced HCC. Early-phase studies of nivolumab and pembrolizumab provided proof of antitumor activity, although objective responses were observed in only approximately 15-20% of previously treated patients (5, 6). Subsequent phase III trials showed that atezolizumab plus bevacizumab and durvalumab plus a single priming dose of tremelimumab (the STRIDE regimen) prolonged overall survival (OS) compared with sorafenib and established immune-based combinations as first-line treatment options for unresectable HCC (7, 8). However, primary resistance remains common, and some patients who initially achieve disease control later develop progression (5–8). These findings indicate that PD-1/PD-L1 or CTLA-4 blockade is insufficient to achieve sustained tumor control in many patients, suggesting that additional immune-evasion mechanisms remain active in HCC (5–8).

Studies of the HCC immune microenvironment have begun to identify mechanisms that may contribute to immune escape. Single-cell and paired-tissue studies have shown that HCC is accompanied by broad changes in both lymphoid and myeloid cell populations, including impaired natural killer (NK)-cell cytotoxicity (9, 10). These findings suggest that tumor immune escape may involve mechanisms that regulate several immune-cell populations. Cell-surface glycosylation is one such mechanism and is of particular interest. Among these glycan changes, altered terminal sialylation is particularly relevant to immune regulation. Studies using human HCC tissues and experimental models have found changes in sialyltransferase expression and terminal sialylation during HCC development (11–14). The biological effects of these changes vary according to the enzyme involved, the glycan structure produced, and the cellular context. They have been associated with immune suppression, tumor invasion, receptor signaling, and, in some settings, reduced metastatic capacity (11–14). Moreover, terminal sialoglycans are particularly relevant because they can be recognized by sialic acid-binding immunoglobulin-like lectins (Siglecs) expressed on immune cells (15, 16). Many inhibitory Siglecs contain immunoreceptor tyrosine-based inhibitory motifs (ITIMs). After a Siglec binds its glycan ligand, these motifs recruit Src homology 2 domain-containing protein tyrosine phosphatases 1 and 2 (SHP-1 and SHP-2) and reduce activating signals in lymphoid and myeloid cells (15). In other words, through this receptor-glycan interaction, tumor-associated sialylation may weaken immune-cell activation and contribute to immune escape. Therefore, the sialylation-Siglec system may act as a glycan-based immune checkpoint.

HCC-specific studies have begun to provide evidence for distinct components of this glyco-immune checkpoint model. Siglec-9-dependent interactions have been linked to impaired intratumoral NK-cell surveillance, whereas Siglec-10^hi^ macrophages have been associated with a suppressive myeloid environment and dysfunctional CD8^+^ T-cell responses (17, 18). Separately, acquired sorafenib resistance has been associated with ST3 β-galactoside α-2,3-sialyltransferase 1 (ST3GAL1)-dependent remodeling of Siglec-binding ligands and reduced susceptibility to NK-cell-mediated killing (19). Together, these findings suggest that altered tumor sialylation may affect several immune-cell populations and may be further reshaped under therapeutic pressure. However, because these observations were obtained from different clinical cohorts and experimental systems, they do not yet establish a single causal sequence linking tumor sialylation, Siglec engagement, immune-cell dysfunction, and treatment resistance (17–19). An important unresolved question is whether the same mechanisms enable residual HCC cells to evade immune clearance after curative-intent treatment and thereby contribute to postoperative recurrence. This possibility remains a testable translational hypothesis (17–19). Accordingly, this review examines sialylation-Siglec glyco-immune checkpoints in HCC, with a focus on NK-cell dysfunction, Siglec-positive macrophage states, therapeutic resistance, and their potential relevance to postoperative recurrence.

## 2 Literature search approach

To prepare this narrative review, PubMed and Web of Science Core Collection were searched from database inception to July 20, 2026. Search terms included combinations of “hepatocellular carcinoma”, “liver cancer”, “sialylation”, “sialic acid”, “sialyltransferase”, “Siglec”, “natural killer cell”, “macrophage”, “immunotherapy”, “drug resistance”, “recurrence”, “circulating tumor DNA”, and “molecular residual disease”. Reference lists of relevant articles were also screened. Priority was given to original studies in HCC. Evidence from other tumor types was included when it provided relevant mechanistic or therapeutic support. Evidence was evaluated according to HCC specificity, use of human samples, functional validation, spatial evidence, clinical association, and therapeutic intervention.

## 3 Aberrant sialylation and the HCC glyco-code

### 3.1 Glycan remodeling and Siglec recognition

Aberrant glycosylation is a regulated feature of the HCC phenotype. Glycans affect protein stability, receptor signaling, cell-matrix adhesion, immune recognition, and treatment sensitivity (20, 21). Unlike proteins, glycan structures are not directly encoded by a genetic template. They are produced through the combined effects of glycosyltransferases and glycosidases, nucleotide-sugar availability, Golgi organization, and the identity and trafficking of glycoprotein and glycolipid carriers (20, 21). These factors interact with one another. Similar levels of glycosyltransferase transcripts can produce different cell-surface glycan patterns, while a similar total amount of sialic acid can be distributed across different molecular carriers (20, 21). For this reason, enzyme expression or total glycan abundance alone provides only a partial view of the HCC glyco-code (20, 21).

Terminal sialylation is especially important because sialic acids are often located at the outer ends of cell-surface glycans. In this position, they can influence membrane organization, receptor accessibility, cell adhesion, and lectin recognition (15, 22). Their biological effects depend on several features, including abundance, α2,3 or α2,6 linkage, the underlying glycan structure, chemical modification, and the protein or lipid carrier on which they are displayed (15, 22). Some sialylated structures can bind Siglecs on immune cells and convert tumor glycan changes into inhibitory immune signals (15). Thus, functional Siglec-ligand formation requires more than an overall increase in sialylation. It also depends on a compatible glycan epitope, an appropriate protein or lipid carrier, sufficient ligand density, and accessible presentation at the cell surface (15, 22). This point is also important for experimental interpretation. Linkage-selective lectins detect broad classes of glycans, whereas Siglec probes fused to the fragment crystallizable (Fc) region of an immunoglobulin indicate whether cell-surface glycans can bind a particular Siglec. Neither method alone identifies the exact glycan structure and its molecular carrier. Therefore, structural glycomics and site-specific glycoproteomics are needed to define glycan structures and, for glycoproteins, connect them to peptide sequences and glycosylation sites (15, 22, 23).

Chemical modifications of sialic acids provide an additional level of control over receptor recognition. For example, 9-O-acetylation markedly reduced the binding of CD22 (Siglec-2) to α2,6-sialylated glycans, whereas enzymatic removal of the acetyl groups increased recognition of cell-surface ligands (24). This finding shows that a sialylated glycan can remain present while its recognition by a Siglec is reduced. O-acetyl groups can also migrate between positions on the sialic acid side chain under commonly used analytical conditions, making their preservation relevant to structural analysis (25). Therefore, characterization of HCC Siglec ligands should consider sialic acid modifications alongside linkage and carrier identity, with their effects established for the receptor under study.

Studies of human tissues show that sialylation changes extensively during the development of HCC, but the pattern differs among tumors (11, 26–28). Dall’Olio et al. reported that ST6 β-galactoside α-2,6-sialyltransferase 1 (ST6GAL1) activity and mRNA expression increased in some tumors and decreased in others. Sambucus nigra agglutinin-reactive α2,6-sialylated glycoconjugates were increased in many specimens but markedly reduced in a minority, and substantial variation was also present within individual tumors (11). In a study of 19 resected HCCs, lower tumor ST6GAL1 activity was associated with a higher circulating proportion of monosialylated α-fetoprotein (AFP) and poorer tumor differentiation (26). In another study of 35 matched tumor and nontumor pairs, CD75s-type α2,6-sialylated gangliosides and iso-CD75s-type α2,3-sialylated gangliosides were increased in only 35% and 41% of tumors, respectively. ST6GAL1 transcript levels were unchanged, whereas ST3 β-galactoside α-2,3-sialyltransferase 6 (ST3GAL6) expression was reduced (27). Intact glycopeptide profiling of 20 paired hepatitis B virus (HBV)-related HCC samples identified reduced sialylated biantennary N-glycans in both AFP groups and increased sialylated triantennary glycans only in the low-AFP group. However, because samples were pooled within AFP strata, the study could not assess variation between individual patients (28). Together, these studies show that HCC sialylation differs according to glycan class, molecular carrier, and tumor context, and that sialyltransferase transcript levels do not fully predict the final surface glycan pattern (11, 26–28).

Complementing these biochemical and glycoproteomic studies, Tang et al. integrated single-cell RNA sequencing of 32,247 cells from human HCC samples with bulk-transcriptomic analyses. Malignant hepatocyte states showed distinct gene-expression-derived sialylation scores, and phosphoglycerate mutase 2 (PGAM2) expression correlated positively with the inferred sialylation program. A nine-gene PGAM2-sialylation-related signature further stratified OS and immune contexture (29). This study therefore links transcriptional heterogeneity in sialylation-related pathways with clinically relevant risk patterns. Its evidence is positioned at the transcriptomic and prognostic-association level, with glycan-resolved and receptor-level studies representing the next step toward identifying the underlying structures, molecular carriers, and Siglec activity.

Experimental studies of ST6GAL1 further show that the effects of α2,6-sialylation depend on the modified substrate and the biological process being studied. Enforced ST6GAL1 expression increased CD147 and matrix metalloproteinase levels, reduced CD8^+^ T-cell proliferation, altered cytokine production, and decreased intratumoral CD8^+^ T-cell infiltration in hepatocarcinoma models (12). However, the sialylated carrier responsible for these immune effects or the immune lectin that recognized it were not identified in this study. The results support an immunoregulatory role for ST6GAL1 without defining a specific ST6GAL1-Siglec pathway (12). In contrast, Zou et al. (13) found that higher ST6GAL1 expression and surface α2,6-sialylation were associated with lower metastatic potential, and that ST6GAL1 overexpression suppressed invasion and metastasis. Metabolic-labeling glycoproteomics identified melanoma cell adhesion molecule (MCAM) as an important carrier. Increased α2,6-sialylation of MCAM weakened galectin-3 binding and reduced MCAM abundance at the cell surface (13). These results describe different biological settings and endpoints. Together, they show that the effects of ST6GAL1 depend on glycan linkage, carrier identity, cell type, tumor state, and the specific phenotype being measured (11–13, 26).

Other sialyltransferases add further complexity beyond ST6GAL1-dependent N-glycans. ST3GAL6 promoted HCC-cell proliferation, migration, and invasion through protein kinase B (AKT)-mechanistic target of rapamycin (mTOR) signaling and mediated part of the tumor-suppressive effect of miR-26a (30). However, the study did not define the α2,3-sialylated structures produced by ST3GAL6 or identify the immune receptors that might recognize them. ST6 N-acetylgalactosaminide α-2,6-sialyltransferase 4 (ST6GALNAC4) was increased in HCC tissues and promoted tumor growth and invasion (14). Man et al. (14) linked ST6GALNAC4 to altered glycosylation of transforming growth factor β receptor 2 (TGFBR2), increased TGFBR2 abundance, stronger transforming growth factor β (TGF-β) signaling, and a proposed interaction between T-antigen, galectin-3, and tumor-associated macrophages. However, the exact TGFBR2 glycoform was not structurally identified, and Siglec involvement was not examined. These findings connect O-glycan remodeling with tumor signaling and changes in immune-cell composition, while leaving the specific receptor-glycan mechanism unresolved (14).

Therapy may also change the HCC glyco-code. Sorafenib-resistant HCC cells showed increased ST3GAL1 expression and greater display of surface ligands capable of binding Siglecs (19). This finding provides tumor-side evidence that treatment pressure can alter sialylation and potentially change how HCC cells interact with immune cells. The immune and clinical consequences of this process are discussed in Section 7.1.

Taken together, current evidence supports a stepwise model in which enzyme expression influences the HCC glyco-code but does not determine it on its own (11–15, 19, 22, 26–28, 30). The relevant sequence is: enzyme expression, glycan linkage and underlying structure, molecular carrier, and Siglec binding, with each step shaped by the cellular and spatial setting (11–15, 19, 22, 26–28, 30). Therefore, the central question is not simply whether HCC cells contain more sialic acid. It is which enzymes produce which sialylated structures, on which protein or lipid carriers, and whether these structures can engage Siglecs on immune cells (**Table 1**, **Figure 1**) (11–15, 19, 22, 26–28, 30).

**Table 1:** HCC evidence linking sialylation remodeling to tumor and immune phenotypes.

Feature	Evidence/model	Key HCC finding	Interpretation or unresolved issue	Refs
ST6GAL1: tissue heterogeneity	Normal, cirrhotic and HCC tissue; resected HCC n = 19; matched tumor/non-tumor n = 35	Activity, transcript and α2,6 reactivity varied within and across tumors. Lower activity was associated with a higher circulating proportion of monosialylated AFP and poorer differentiation; transcript did not consistently predict glycan phenotype.	Most carriers remain unresolved. Direct glycan measurement is preferable to inference from enzyme expression.	(11, 26, 27)
ST6GAL1: immune phenotype	HCC cells and experimental models	Overexpression increased α2,6 sialylation, CD147 and MMPs; reduced CD8^+^ T-cell proliferation; altered cytokines; and decreased intratumoral CD8^+^ T cells.	Supports an immunoregulatory phenotype; the responsible carrier and lectin pathway remain unassigned.	(12)
ST6GAL1-MCAM	HCC cells and metastasis models	MCAM α2,6 sialylation weakened galectin-3 binding and reduced surface MCAM. Higher ST6GAL1 expression and α2,6 sialylation were associated with lower metastatic potential; overexpression suppressed invasion and metastasis.	Phenotypic direction depends on carrier, binding partner and endpoint.	(13)
ST3GAL6	Matched human tissue and HCC cells	Transcript was reduced in one tissue cohort, whereas experimental ST3GAL6 promoted proliferation, migration and invasion through AKT-mTOR and mediated part of the tumor-suppressive effect of miR-26a.	α2,3 products and carriers were not assigned; transcript, structure and phenotype require same-sample analysis.	(27, 30)
ST6GALNAC4	Human HCC tissue, cells and tumor models	ST6GALNAC4 was increased in HCC and promoted growth and invasion with greater TGFBR2 abundance, stronger TGF-β signaling and altered immune composition.	TGFBR2 is a candidate O-glycan carrier; the exact glycoform and proposed T-antigen-galectin-3-TAM interface require structural validation.	(14)
Site-specific N-glycosylation	20 paired HBV-related HCC/non-tumor samples, pooled by AFP stratum	Compared with matched non-tumor liver, sialylated biantennary N-glycans decreased in both AFP strata, whereas sialylated triantennary glycans increased only in the low-AFP stratum.	Pooling obscured individual variation; future studies need individual, multi-region glycoproteomics.	(28)
PGAM2-sialylation-related transcriptomic signature	Single-cell RNA sequencing of 32,247 cells from human HCC samples; bulk-transcriptomic prognostic modeling; *in vitro* AGRN knockdown	Malignant hepatocyte states differed in gene-expression-derived sialylation scores. PGAM2 expression correlated with the inferred score, and a nine-gene signature stratified OS and immune contexture. AGRN, one signature component, supported HCC-cell proliferation and invasion.	Positions the evidence at the transcriptomic and prognostic-association level; functional AGRN experiments support the biological relevance of one signature component, while glycan-resolved, carrier-specific, and Siglec-functional studies remain the next step.	(29)
ST3GAL1: sorafenib-resistant models and clinical association	Glycan analyses in sorafenib-resistant Huh7/HepG2 cells; functional assays in EGFR-expressing HCC cells, including resistant Huh7 cells; separate stage I-II HCC cohort, n = 107	Sorafenib-resistant cells showed increased ST3GAL1 expression and increased surface binding of Siglec-7-Fc and Siglec-9-Fc probes. ST3GAL1 silencing predominantly reduced Siglec-7-Fc binding and enhanced NK-cell killing and cetuximab-mediated ADCC in the functional models. In the separate cohort, high ST3GAL1 expression was associated with shorter RFS (P = 0.039); the OS difference was not statistically significant.	Supports a functional contribution of ST3GAL1-dependent glycosylation. The relevant glycan-carrier pairs, Siglec dependence, and treatment-specific predictive value require further validation.	(19)

ADCC, antibody-dependent cellular cytotoxicity; AFP, α-fetoprotein; AGRN, agrin; Fc, fragment crystallizable region; AKT, protein kinase B; EGFR, epidermal growth factor receptor; HBV, hepatitis B virus; HCC, hepatocellular carcinoma; MCAM, melanoma cell adhesion molecule; miR-26a, microRNA-26a; MMPs, matrix metalloproteinases; mTOR, mechanistic target of rapamycin; NK, natural killer; OS, overall survival; PGAM2, phosphoglycerate mutase 2; RFS, recurrence-free survival; RNA, ribonucleic acid; Siglec, sialic acid-binding immunoglobulin-like lectin; ST3GAL1, ST3 β-galactoside α-2,3-sialyltransferase 1; ST3GAL6, ST3 β-galactoside α-2,3-sialyltransferase 6; ST6GAL1, ST6 β-galactoside α-2,6-sialyltransferase 1; ST6GALNAC4, ST6 N-acetylgalactosaminide α-2,6-sialyltransferase 4; TAM, tumor-associated macrophage; TGF-β, transforming growth factor β; TGFBR2, transforming growth factor β receptor 2.

**Figure 1:**
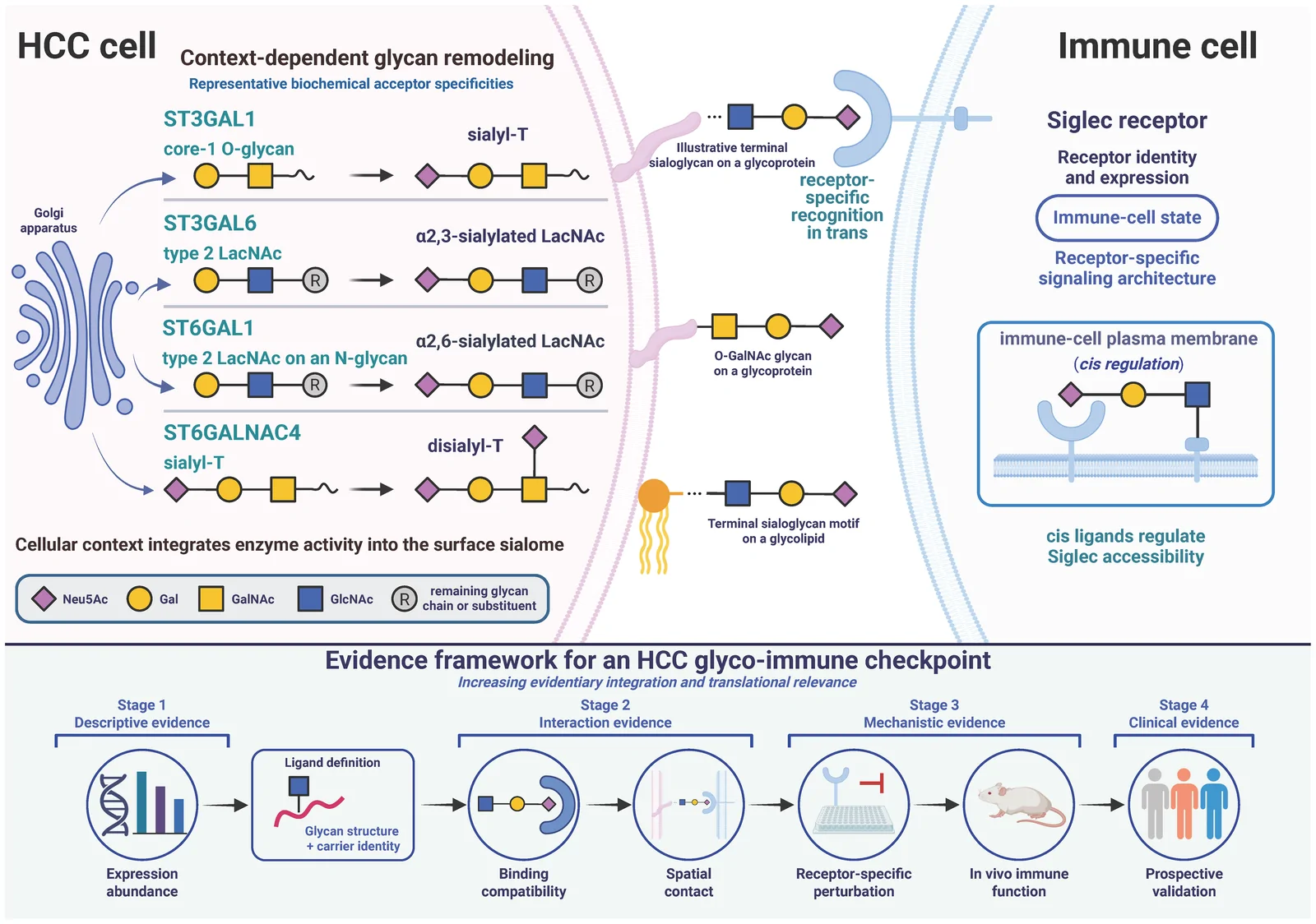
Context-dependent glycan remodeling and an evidence framework for a sialylation-Siglec glyco-immune checkpoint in HCC. The upper panel shows how sialyltransferases in the Golgi apparatus of HCC cells act on representative glycan acceptors. ST3GAL1 converts the core 1 O-glycan to sialyl-T. ST3GAL6 generates α2,3-sialylated LacNAc, whereas ST6GAL1 generates α2,6-sialylated LacNAc on N-glycans. ST6GALNAC4 converts sialyl-T to disialyl-T. Enzyme activity, substrate availability, glycan structure, chemical modifications, carrier identity, and cellular context together shape the cell-surface sialome. Terminal sialoglycans on glycoproteins or glycolipids may bind Siglecs in trans on adjacent immune cells. Cis ligands on the same immune-cell surface can regulate Siglec accessibility. The functional outcome also depends on Siglec identity and expression, immune-cell state, and receptor-specific signaling pathways. The lower panel presents a four-stage framework for evaluating an HCC glyco-immune checkpoint. Descriptive evidence shows changes in receptor or ligand abundance. Interaction evidence defines the glycan structure and carrier and demonstrates binding compatibility and spatial receptor-ligand contact. Mechanistic evidence evaluates changes in signaling or immune function after intervention, with receptor-specific perturbation and appropriate *in vivo* studies strengthening causal attribution. The final stage emphasizes prospective clinical validation. Ellipses indicate omitted or unspecified glycan structures. Gal, galactose; GalNAc, N-acetylgalactosamine; GlcNAc, N-acetylglucosamine; HCC, hepatocellular carcinoma; LacNAc, N-acetyllactosamine; Neu5Ac, N-acetylneuraminic acid; R, remaining glycan chain or substituent; Siglec, sialic acid-binding immunoglobulin-like lectin; ST3GAL1, ST3 β-galactoside α-2,3-sialyltransferase 1; ST3GAL6, ST3 β-galactoside α-2,3-sialyltransferase 6; ST6GAL1, ST6 β-galactoside α-2,6-sialyltransferase 1; ST6GALNAC4, ST6 N-acetylgalactosaminide α-2,6-sialyltransferase 4. Created in BioRender. Zhang, K (2026). https://BioRender.com/en20usb.

### 3.2 Biophysical effects of the tumor glycocalyx

Sialylation also contributes to the physical properties of the cell surface. At physiological pH, N-acetylneuraminic acid (Neu5Ac) carries a negatively charged carboxylate group, contributing to surface charge and repulsive interactions between cells (22). Direct force measurements showed that polysialylation of neural cell adhesion molecule increased intermembrane repulsion sufficiently to overcome both neural cell adhesion molecule-mediated and cadherin-mediated adhesion at physiological ionic strength (31). This provides a physical mechanism through which sialylated glycans can weaken cell-cell contacts. The broader organization of the glycocalyx also affects membrane shape. Shurer et al. demonstrated that the size and surface density of mucin polymers and other long-chain glycocalyx components generated forces that promoted membrane curvature, tubular protrusions, and vesicle formation (32). These findings identify surface charge, glycan-chain dimensions, and molecular crowding as distinct contributors to cell-surface mechanics, providing a basis for examining how altered sialylation affects tumor-cell detachment and migration.

A bulky glycocalyx can also strengthen localized cell-matrix adhesions. Paszek et al. showed that large surface glycoproteins restricted integrin binding outside existing adhesion sites while promoting integrin clustering, focal adhesion assembly, and growth and survival signaling at established contacts (33). Thus, reduced access to the cell surface can coexist with stronger signaling at selected adhesion sites. Extending this mechanism *in vivo*, Woods et al. found that increasing glycocalyx thickness enhanced integrin-focal adhesion kinase signaling and cell-cycle progression, promoting the growth of disseminated mammary tumor cells in the lungs (34). These studies establish that glycocalyx architecture can support tumor progression through physical regulation of adhesion and signaling. In HCC, the ST6GAL1-MCAM findings discussed above further show that the direction of an adhesion-related phenotype depends on the modified carrier and its binding partners (13). Therefore, glycocalyx mechanics provides a complementary framework for investigating tumor-cell dissemination and subsequent growth alongside Siglec-mediated immune escape. Whether these processes cooperate to support residual HCC cells and postoperative recurrence remains a defined question for future studies.

## 4 Siglec receptors as glyco-immune checkpoints in HCC

Siglecs are expressed mainly on leukocytes. Their functions vary according to the receptor subtype, the immune cell on which they are expressed, and the signaling molecules linked to their cytoplasmic domains (15, 35, 36). Most CD33-related Siglecs contain ITIMs or ITIM-like motifs that recruit SHP-1 and/or SHP-2 after ligand binding, whereas other family members, including Siglec-15, associate with adaptors such as DNAX activation protein 12 (DAP12) (15, 35–38). Siglec function is also influenced by cis ligands on the same immune-cell surface, which can mask the receptor or affect its organization and accessibility to trans ligands on neighboring cells (15, 36). The early identification of Siglec-7 as an inhibitory receptor on NK cells provided direct evidence that glycan recognition can suppress NK-cell cytotoxicity (39, 40). Therefore, Siglecs form a diverse receptor family, and each receptor must be evaluated according to its cellular location, ligand recognition, and signaling pathway (15, 35, 36).

Evidence for an active sialylation-Siglec checkpoint in HCC can be considered at four levels. The first is descriptive evidence showing changes in receptor or ligand abundance. The second is interaction evidence showing compatible ligand binding or close receptor-ligand contact within tissue. The third is mechanistic evidence showing that genetic, biochemical, or pharmacological disruption changes receptor signaling or immune-cell function. The fourth is clinical evidence linking a functionally defined pathway to treatment response, recurrence, or survival in well-characterized patient cohorts. Progression through these levels requires more than receptor expression. Checkpoint activity also depends on a compatible glycan structure and carrier, adequate ligand density, surface accessibility, receptor availability, and an immune-cell state that permits signaling (15, 16, 36, 41–44). However, no HCC pathway has yet combined all of the following: identification of the endogenous glycan and its carrier, direct evidence of receptor activation within tumor tissue, receptor-specific intervention in an HCC model with compatible receptor-ligand pairs and relevant immune compartments, and prospective clinical validation. Accordingly, receptor abundance alone should not be interpreted as evidence of an active glyco-immune checkpoint (**Figure 1**) (17–19, 45, 46).

This evidence framework is especially relevant in HCC because NK-cell and macrophage populations undergo marked changes within the tumor immune microenvironment (9, 10). Among the Siglec pathways studied so far, the Siglec-9-NK-cell axis has the strongest and most complete HCC-specific support. Available studies have examined receptor expression, spatial receptor-ligand relationships, associations with clinical outcomes, and functional interruption in preclinical models (17). The Siglec-10-macrophage axis is also supported by analyses of human HCC tissues, postoperative outcomes, and ex vivo intervention experiments, whereas its endogenous ligand in HCC and its suitability as an *in vivo* therapeutic target remain to be defined (18). Evidence for Siglec-7 is more fragmented. Reduced Siglec-7 expression on circulating NK cells has been associated with impaired NK-cell function (45). Moreover, sorafenib-resistant HCC cells have been shown to display ST3GAL1-dependent ligands capable of binding Siglec-7 (19). These findings identify relevant changes on both the immune-cell and tumor-cell sides. However, direct spatial contact between Siglec-7-positive NK cells and these ligands has not been demonstrated in human HCC, and receptor-specific causal testing is still lacking (19, 45).

Siglec-15 should be regarded as boundary evidence. SIGLEC15 expression was increased in HCC and showed an inverse association with PD-L1 expression. Genetic deletion or antibody treatment also improved CD8^+^ T-cell viability and cytotoxicity in experimental systems (46). These findings support an immune-regulatory role for Siglec-15 in HCC. However, Siglec-15 signals through DAP12, and the relevant sialylated ligand, its molecular carrier, and the contribution of glycan recognition were not identified (38, 46). The available evidence supports Siglec-15 as an immune-regulatory Siglec-family receptor, while its role as a defined glycan-dependent checkpoint remains uncertain (38, 46).

Overall, current evidence places the Siglec-9-NK-cell and Siglec-10-macrophage axes at the center of the HCC sialylation-Siglec framework (17, 18). Siglec-7 provides complementary evidence linking NK-cell dysfunction with treatment-induced changes in tumor ligands (19, 45). Siglec-15 remains relevant as supporting evidence from the wider Siglec family (**Table 2**). Sections 5 and 6 examine the NK-cell and macrophage pathways in greater detail, while Section 7 discusses their possible roles in treatment resistance and postoperative recurrence.

**Table 2:** Evidence maturity of candidate Siglec-centered immune-regulatory axes in HCC.

Axis	Compartment	Evidence maturity	Key HCC evidence	Decisive gap	Refs
Siglec-9-NK	Tumor-infiltrating NK cells	D + I + M + C (retrospective)	Higher intratumoral Siglec-9 expression was associated with shorter survival; greater abundance of cells bearing Siglec-9-compatible ligands in close proximity to Siglec-9^+^ NK cells was associated with recurrence and shorter survival. MTX-3937 reduced phosphorylation of Siglec-9, SHP-1 and SHP-2 and increased NK-cell degranulation, cytokine production and cytotoxicity; MTX-3937-treated NK-92 cells suppressed hepatoma xenografts.	Define the endogenous glycan-carrier pair; establish compound selectivity and receptor dependence in models with compatible receptor-ligand pairs; validate the spatial marker prospectively.	(17)
Siglec-10-TAM	Predominantly CD68^+^ TAMs	D + M (ex vivo ligand sequestration) + C (retrospective)	In 235 resected HCCs (115 discovery; 120 validation), higher intratumoral Siglec-10^+^ cell abundance was associated with worse OS (HR 1.691; P = 0.004) and RFS (HR 1.577; P = 0.008). Soluble Siglec-10-Fc ligand sequestration reduced macrophage suppressive markers, restored CD8+ T-cell effector features and enhanced pembrolizumab-associated tumor-cell apoptosis ex vivo.	Resolve the ligand-carrier pair and spatial interface; establish Siglec-10 dependence through selective blockade, deletion and rescue; confirm *in vivo* activity and added prognostic value beyond TAM abundance.	(18)
Siglec-7-NK	Circulating NK subsets; proposed tumor interface	D + I (probe binding) + M (ligand-directed) + C (separate datasets)	Siglec-7-negative circulating NK cells were functionally weaker. Separately, ST3GAL1 silencing reduced Siglec-7-Fc probe binding and enhanced NK-cell killing and ADCC in resistant HCC cells.	Integrate receptor, native ligand/carrier, spatial contact, proximal signaling and NK function in the same HCC system.	(19, 45)
Siglec-15	Tumor and immune compartment; CD8^+^ T-cell effects	D + M	SIGLEC15 expression was increased and inversely associated with PD-L1; deletion or antibody treatment improved CD8^+^ T-cell viability and cytotoxicity in experimental systems.	Define cellular source and ligand dependence; separate glycan-dependent signaling from broader Siglec-15 biology.	(38, 46)
Siglec-5-macrophage	THP-1 macrophages in HepG2 coculture	M (model)	Under hypoxia, Siglec-5 overexpression favored an arginase-1-high state through altered SHP-2-SYK signaling.	Confirm endogenous expression, signaling and phenotype in primary human TAMs and etiologically relevant *in vivo* HCC.	(47)

D, I, M, and C indicate descriptive, interaction, mechanistic, and clinical evidence, respectively. These categories describe evidence maturity and do not represent a quantitative score. Qualifiers specify the intervention type and clinical study context; M does not by itself establish receptor specificity. A plus sign between evidence codes indicates that more than one type of evidence is available. The final column identifies the main validation gap for each candidate axis. MTX-3937 is the small molecule investigated as a Siglec-9 inhibitor in the cited study.

ADCC, antibody-dependent cellular cytotoxicity; Fc, fragment crystallizable region; HCC, hepatocellular carcinoma; HR, hazard ratio; NK, natural killer; OS, overall survival; PD-L1, programmed death-ligand 1; RFS, recurrence-free survival; SHP-1/2, Src homology 2 domain-containing protein tyrosine phosphatases 1 and 2; Siglec, sialic acid-binding immunoglobulin-like lectin; SIGLEC15, sialic acid-binding immunoglobulin-like lectin 15 gene; ST3GAL1, ST3 β-galactoside α-2,3-sialyltransferase 1; SYK, spleen-associated tyrosine kinase; TAM, tumor-associated macrophage.

## 5 NK-cell dysfunction and the sialylation-Siglec axis

NK cells are key effectors of hepatic immune surveillance. By integrating activating and inhibitory signals, they can eliminate stressed, infected, or transformed cells without prior antigen sensitization (48). The healthy adult liver contains abundant NK cells, including recirculating CD56^dim^ cells and a long-lived tissue-resident CD56^bright^Eomes^hi^T-bet^lo^CXCR6^+^ population (49, 50). The coexistence of circulating and resident NK-cell populations indicates that hepatic immune surveillance depends on both newly recruited cells and NK cells maintained locally within the liver (49, 50).

HCC markedly alters this NK-cell compartment. Single-cell studies have shown depletion and substantial variation in intratumoral T- and NK-cell populations among patients (9). Paired analyses of tumor and adjacent liver tissues have also identified fewer tumor-infiltrating NK cells and an increase in poorly cytotoxic CD56^bright^CD16^−^ cells. These cells expressed less NKG2D, more NKG2A, and showed impaired degranulation and reduced cytokine production (9, 10). An expanded CD49a^+^Eomes^+^ NK-cell population also displayed weak degranulation and produced proangiogenic factors after stimulation (10). These findings suggest that HCC can shift NK cells away from effective tumor surveillance and toward dysfunctional, regulatory-like, or proangiogenic states (9, 10).

Within this altered NK-cell landscape, the Siglec-9-ligand interaction currently has the strongest HCC-specific support as a glyco-immune checkpoint. Using multiplex immunofluorescence, Xiao et al. found increased Siglec-9 expression on tumor-infiltrating NK cells, while Siglec-7 did not show the same increase (17). Higher Siglec-9 expression was associated with shorter survival. More importantly, a higher abundance of cells bearing Siglec-9-compatible ligands in close spatial proximity to Siglec-9^+^ NK cells was associated with a greater likelihood of recurrence and shorter survival (17). This finding highlights the importance of the local receptor-ligand interface and suggests that Siglec-9 expression alone may provide an incomplete measure of checkpoint activity (17). The same study also provided functional evidence for this pathway. MTX-3937, a small-molecule Siglec-9 inhibitor, reduced phosphorylation of Siglec-9, SHP-1, and SHP-2 and increased NK-cell degranulation, cytokine production, and cytotoxicity (17). Adoptive transfer of MTX-3937-treated NK-92 cells also suppressed hepatoma xenograft growth and prolonged survival in immunodeficient mice (17). These experiments show that the pathway can be disrupted in preclinical systems. However, its receptor specificity, systemic safety, and ability to prevent HCC recurrence still require further testing.

The findings for Siglec-7 require a different interpretation. In peripheral blood from 35 patients with HCC and 63 healthy controls, Tao et al. (45) observed fewer Siglec-7^+^ NK cells in HCC, while Siglec-7^−^ NK cells expressed fewer activating receptors and showed weaker cytokine production and cytotoxicity than Siglec-7^+^ cells. These results are compatible with the known inhibitory function of ligand-engaged Siglec-7 in tumor models because the two studies examined different biological settings (41). Therefore, reduced Siglec-7 expression in blood may reflect altered NK-cell differentiation or reduced functional fitness. It does not directly indicate the level of Siglec-7 checkpoint signaling inside the tumor (41, 45). In summary, receptor abundance, NK-cell functional state, and local ligand engagement should be assessed as separate features.

Tumor-side evidence adds another part to the Siglec-7 model. Sorafenib-resistant HCC cells showed ST3GAL1-dependent remodeling of surface glycans, increased display of Siglec-compatible ligands, and reduced susceptibility to NK-cell-mediated killing (19). These findings suggest that treatment pressure can alter tumor glycans in a way that weakens NK-cell clearance. However, the available studies have not directly connected ST3GAL1-dependent ligands on resistant HCC cells with Siglec-7 activation on tumor-infiltrating NK cells. The detailed resistance experiments and clinical findings are discussed in Section 7.1.

Siglec engagement is unlikely to be the only determinant of NK-cell dysfunction in HCC. Studies involving RAC1-engineered NK-92 cells, disruption of TGF-β signaling in HCC-directed induced pluripotent stem cell (iPSC)-derived NK cells, and Nur77 activation collectively indicate that NK-cell activity is also constrained by intrinsic functional fitness, suppressive cytokine signaling, and metabolic stress (51–53). These studies do not establish interactions with Siglec pathways, but they show that restoring target recognition alone may be insufficient within a multilayered suppressive microenvironment (51–53).

Overall, current evidence most strongly supports a functional Siglec-9 checkpoint in tumor-infiltrating NK cells (17). Evidence for Siglec-7 comes from two separate observations: reduced Siglec-7 expression on circulating NK cells and ST3GAL1-dependent formation of Siglec-7-compatible ligands on sorafenib-resistant HCC cells (19, 45). Spatial proximity between receptor-bearing NK cells and ligand-bearing tumor cells is biologically relevant, but receptor activation should also be confirmed through measures such as Siglec, SHP-1, or SHP-2 phosphorylation and NK-cell-specific functional assays (17). Clinical studies are still needed to determine whether Siglec-directed treatment can restore NK-cell surveillance, improve treatment response, or reduce postoperative recurrence in HCC (**Figure 2**) (17, 19, 45).

**Figure 2:**
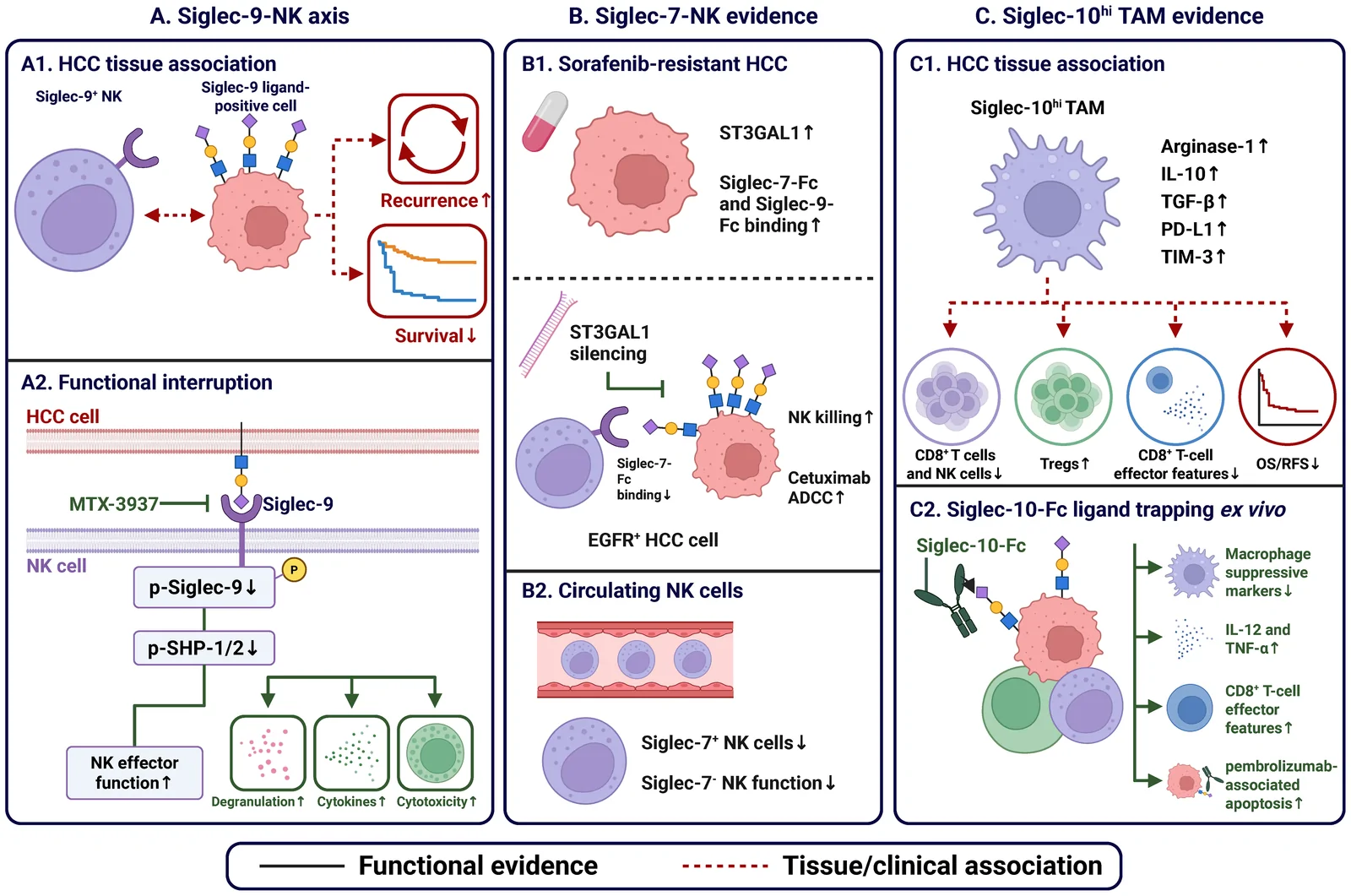
Cell-specific evidence for Siglec-associated immune regulation in HCC. **(A)** summarizes tissue and functional evidence for the Siglec-9-NK-cell axis. Close spatial proximity between Siglec-9^+^ NK cells and Siglec-9 ligand-positive cells is associated with increased recurrence and shorter survival. MTX-3937 reduces Siglec-9-SHP-1/2 signaling and increases NK-cell degranulation, cytokine production, and cytotoxicity. **(B)** presents two separate findings related to Siglec-7. Sorafenib-resistant HCC cells show increased ST3GAL1 expression and increased binding of Siglec-7-Fc and Siglec-9-Fc. ST3GAL1 silencing reduces Siglec-7-Fc binding and increases direct NK-cell killing and cetuximab-mediated ADCC against EGFR-expressing HCC cells. Circulating Siglec-7^+^ NK cells are reduced in HCC, whereas Siglec-7^-^ NK cells show impaired function. **(C)** summarizes tissue and ex vivo evidence related to Siglec-10^hi^ TAMs. These cells are associated with an immunosuppressive phenotype, reduced CD8^+^ T-cell and NK-cell infiltration, increased Tregs, impaired CD8^+^ T-cell activity, and shorter OS and RFS. Ex vivo ligand trapping with Siglec-10-Fc reduces suppressive macrophage markers and increases IL-12, TNF-α, CD8^+^ T-cell effector features, and pembrolizumab-associated tumor-cell apoptosis. Solid lines indicate functional intervention findings. Receptor-directed and ligand-directed interventions are distinguished by their labels. Dashed red lines indicate tissue or clinical associations. ADCC, antibody-dependent cellular cytotoxicity; EGFR, epidermal growth factor receptor; Fc, fragment crystallizable region; HCC, hepatocellular carcinoma; IL, interleukin; NK, natural killer; OS, overall survival; PD-L1, programmed death-ligand 1; p-Siglec-9, phosphorylated Siglec-9; RFS, recurrence-free survival; SHP-1/2, Src homology 2 domain-containing protein tyrosine phosphatases 1 and 2; p-SHP-1/2, phosphorylated SHP-1 and SHP-2; Siglec, sialic acid-binding immunoglobulin-like lectin; ST3GAL1, ST3 β-galactoside α-2,3-sialyltransferase 1; TAM, tumor-associated macrophage; TGF-β, transforming growth factor β; TIM-3, T-cell immunoglobulin and mucin domain-containing protein 3; TNF-α, tumor necrosis factor α; Treg, regulatory T cell. Created in BioRender. Zhang, K. (2026) https://BioRender.com/en20usb.

## 6 Siglec-positive tumor-associated macrophages and macrophage-mediated immune suppression

Tumor-associated macrophages (TAMs) are abundant and highly adaptable cells within the HCC microenvironment. Their diversity extends beyond the conventional M1-M2 classification (54). Single-cell and spatial studies have identified several macrophage states that change after treatment. These include TREM2^+^ macrophages associated with reduced CD8^+^ T-cell recruitment or function after transarterial chemoembolization (TACE), as well as PD-L1^+^ macrophage-tumor-cell niches that support stem-like residual HCC cells through TGF-β signaling (9, 55–58). These studies establish macrophages as important organizers of immune exclusion and residual-disease niches (9, 55–58).

Within this heterogeneous compartment, Siglec-10^hi^ TAMs provide the strongest HCC-specific evidence for a candidate macrophage-centered glyco-immune checkpoint. Xiao et al. (18) examined 235 patients who underwent hepatectomy, including a discovery cohort of 115 patients and a validation cohort of 120 patients. Siglec-10^+^ cells were enriched in tumor tissue and were found mainly among CD68^+^ macrophages. Higher intratumoral Siglec-10^+^ cell abundance was independently associated with worse OS (hazard ratio [HR], 1.691; P = 0.004) and recurrence-free survival (RFS) (HR, 1.577; P = 0.008) (18). Siglec-10^hi^ TAMs showed a mixed phenotype. They co-expressed CD206, CD80, CD86, and HLA-DR, while also expressing higher levels of arginase-1, interleukin-10 (IL-10), TGF-β, T-cell immunoglobulin and mucin domain-containing protein 3 (TIM-3), and PD-L1 than Siglec-10^lo^ TAMs (18). RNA sequencing of macrophages sorted from three HCC samples also identified changes in metabolic and immune-response pathways (18). Although the sample size was small, these results suggest that the biological activity of human TAMs cannot be inferred from a few conventional polarization markers alone.

High Siglec-10^hi^ TAM infiltration was associated with fewer intratumoral CD8^+^ T cells and NK cells, more FOXP3^+^ regulatory T cells, and a CD8^+^ T-cell state characterized by lower granzyme B, perforin, interferon-γ (IFN-γ), and IL-2 but higher PD-1, TIM-3, T-cell immunoreceptor with Ig and ITIM domains (TIGIT), lymphocyte activation gene 3 (LAG-3), and CTLA-4 (18). In fresh HCC suspensions, treatment with a soluble Siglec-10-Fc chimera reduced macrophage immunosuppressive markers, increased IL-12 and tumor necrosis factor α (TNF-α), improved CD8+ T-cell effector features, and enhanced pembrolizumab-associated tumor-cell apoptosis ex vivo (18). These findings provide functional evidence that the suppressive immune state can be modified by a ligand-directed intervention. Siglec-10-Fc binds extracellular ligands rather than selectively blocking endogenous Siglec-10. Because glycan recognition can be shared across Siglecs, ligand sequestration may also affect interactions involving other receptors (59). The observed response therefore supports ligand-directed immune modulation, while its dependence on macrophage Siglec-10 remains to be established. Receptor-selective blocking antibodies, SIGLEC10 deletion, and restoration of receptor expression in primary macrophages would help resolve this attribution. Such experiments should connect receptor signaling with macrophage function and downstream T-cell responses.

The endogenous HCC ligand that activates Siglec-10 also remains unresolved. In breast and ovarian cancer, tumor-cell CD24 binds macrophage Siglec-10 and suppresses phagocytosis, providing evidence for a CD24-Siglec-10 “don’t-eat-me” pathway in other tumor types (60). The HCC study did not demonstrate that CD24, or any other defined glycoprotein or glycolipid carrier, was the dominant Siglec-10 ligand (18). Accordingly, defining an HCC-specific pathway will require structural identification of the relevant sialoglycans and their carriers, direct receptor-ligand binding studies, and spatial confirmation within tumor tissue.

Evidence for other macrophage Siglecs is more limited. In a THP-1/HepG2 coculture model, Siglec-5 overexpression promoted an arginase-1-high macrophage state through changes in SHP-2-SYK signaling under hypoxia (47). The responsible ligand was not identified, and the findings have not yet been confirmed in primary human TAMs or immunocompetent HCC models (47).

Taken together, current HCC evidence supports a link between Siglec-10^hi^ TAMs, suppressive myeloid activity, and CD8^+^ T-cell dysfunction (18). Intratumoral Siglec-10^+^ cell abundance is also associated with poorer outcomes after surgery. However, this clinical association alone cannot show that Siglec-10 ligand engagement directly causes recurrence (18). Future studies should identify the relevant HCC ligands, test receptor-specific interventions in models with compatible receptor-ligand pairs and relevant immune compartments, and determine whether spatial contact between ligand-bearing tumor cells and Siglec-10^hi^ TAMs improves recurrence prediction beyond macrophage abundance alone (**Figure 2**). Prediction of treatment benefit should be evaluated separately.

## 7 Therapeutic resistance and postoperative recurrence: from therapy-induced glycan remodeling to a testable molecular residual disease hypothesis

### 7.1 Sorafenib-associated sialylation remodeling and NK-cell escape

The strongest HCC-specific evidence linking sialylation with treatment resistance comes from sorafenib-conditioned models (19). Earlier glycomic studies showed that sorafenib exposure altered protein glycosylation in HCC cells, although the immune effects of these changes were not examined (61). Fan et al. generated sorafenib-resistant Huh7 and HepG2 cells through prolonged dose escalation. Both models showed increased ST3GAL1 expression, reduced peanut agglutinin binding, and increased surface binding of Siglec-7-Fc and Siglec-9-Fc probes (19). Binding was lost with sialic acid-binding-deficient probe mutants, supporting sialic acid-dependent recognition (19). ST3GAL1 silencing predominantly reduced Siglec-7-Fc binding. In functional experiments using epidermal growth factor receptor (EGFR)-expressing HCC cells, including sorafenib-resistant Huh7 cells, silencing increased NK-cell-mediated killing and cetuximab-mediated antibody-dependent cellular cytotoxicity (ADCC) (19). These results establish a functional contribution of ST3GAL1 to reduced susceptibility to NK-cell attack in the tested models (**Figure 3**).

**Figure 3:**
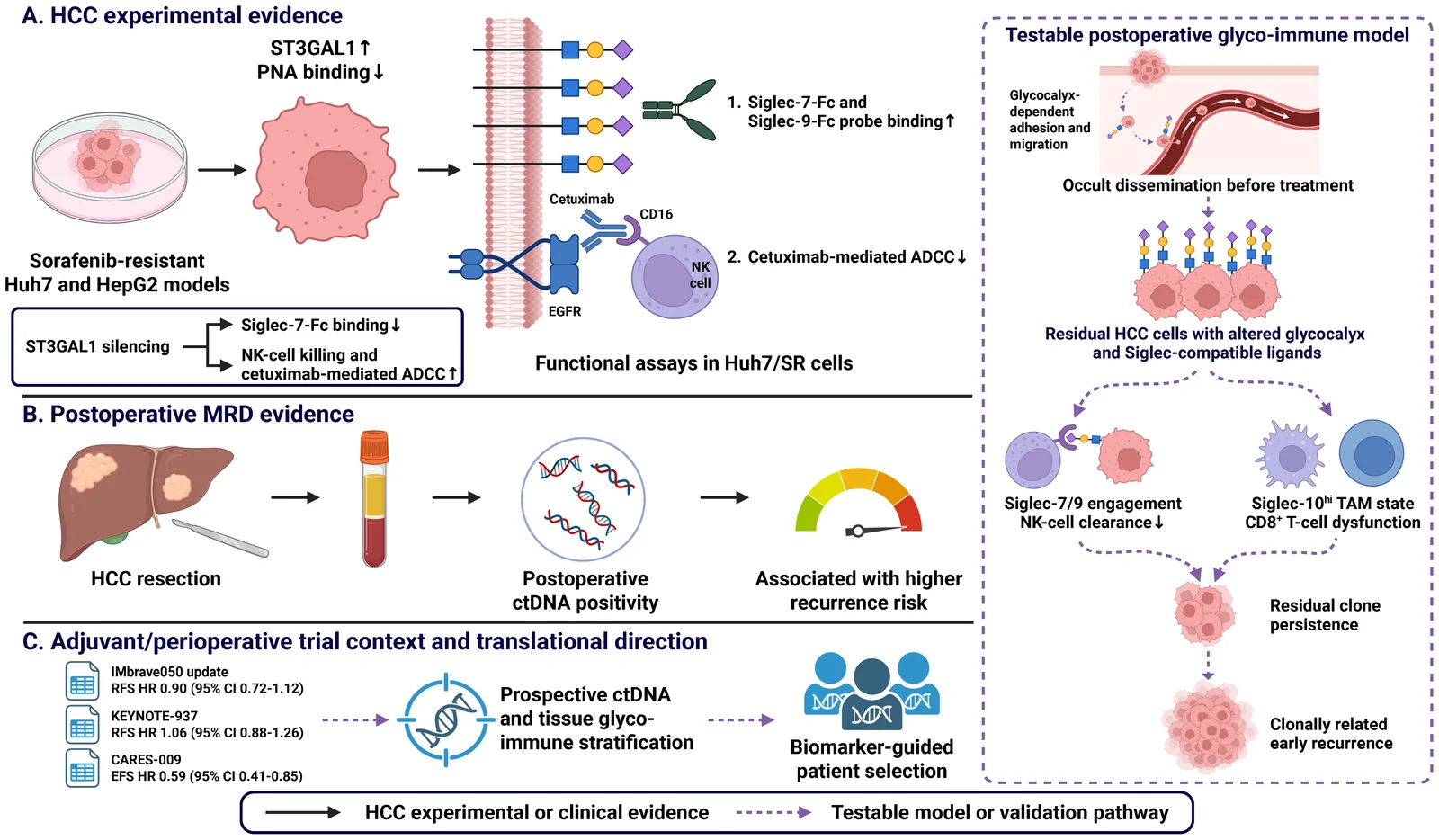
Experimental and clinical evidence informing a testable postoperative glyco-immune model in HCC. **(A)** summarizes glycan changes in sorafenib-resistant Huh7 and HepG2 cells and functional findings in Huh7/SR cells. Increased ST3GAL1 expression is accompanied by reduced PNA binding and increased Siglec-7-Fc and Siglec-9-Fc probe binding. Cetuximab-mediated ADCC is reduced against sorafenib-resistant Huh7 cells compared with control cells. ST3GAL1 silencing reduces Siglec-7-Fc binding and increases direct NK-cell killing and cetuximab-mediated ADCC. Probe-binding and functional changes are shown as separate intervention outcomes. **(B)** shows postoperative ctDNA positivity as a marker of MRD associated with increased recurrence risk after HCC resection. **(C)** places findings from adjuvant and perioperative trials within a proposed validation pathway that combines postoperative ctDNA with tissue glyco-immune features for prospective patient stratification. The model proposes that glycocalyx-dependent adhesion and migration may contribute to occult dissemination before treatment. Residual HCC cells with altered glycocalyx and Siglec-compatible ligands may evade NK-cell clearance through Siglec-7/9 engagement and coexist with a Siglec-10^hi^ TAM state and CD8^+^ T-cell dysfunction. These conditions may support residual clone persistence and clonally related early recurrence. Solid black arrows indicate HCC experimental or clinical evidence. Dashed purple arrows indicate the testable model or proposed validation pathway. ADCC, antibody-dependent cellular cytotoxicity; CI, confidence interval; ctDNA, circulating tumor DNA; EFS, event-free survival; Fc, fragment crystallizable region; HCC, hepatocellular carcinoma; HR, hazard ratio; MRD, molecular residual disease; NK, natural killer; PNA, peanut agglutinin; RFS, recurrence-free survival; Siglec, sialic acid-binding immunoglobulin-like lectin; SR, sorafenib-resistant; ST3GAL1, ST3 β-galactoside α-2,3-sialyltransferase 1; TAM, tumor-associated macrophage. Created in BioRender. Zhang, K. (2026) https://BioRender.com/en20usb.

The loss-of-function results support ST3GAL1 dependence of part of the phenotype, but do not establish that ST3GAL1 is necessary and sufficient for the complete immune-evasion phenotype (19). Gain-of-function and rescue experiments would clarify these roles. Increased Siglec-Fc binding establishes binding capacity, while identification of the endogenous glycoprotein or glycolipid carriers remains a separate step (19, 62). Likewise, testing whether the effect of ST3GAL1 silencing persists after selective disruption of Siglec-7 or Siglec-9 in NK cells would distinguish receptor-dependent inhibition from other consequences of glycan remodeling. Enzyme activity, probe binding, and receptor-dependent immune suppression should therefore be treated as distinct, experimentally connected levels of evidence. Paired tumor samples collected before sorafenib treatment and after progression are needed to determine whether the experimental glycan changes also occur in patients. The clinical analysis in the same study included 107 patients with stage I-II HCC. High ST3GAL1 expression was associated with shorter relapse-free survival (P = 0.039), while the difference in OS was not statistically significant (19). The cutoff for ST3GAL1 expression was selected using recurrence data from the same cohort. In addition, the analysis did not show that treatment caused ST3GAL1 upregulation or that recurrent tumors arose from ST3GAL1-high, highly sialylated clones (19). The reported association between ST3GAL1 and outcomes after immune checkpoint inhibition was also derived from a pooled pan-cancer public dataset, rather than from an HCC-specific treated cohort (19). Therefore, current evidence supports ST3GAL1 as an experimental mediator of NK-cell escape and a candidate biomarker. Its ability to predict sorafenib resistance or immunotherapy response in patients with HCC still requires direct clinical validation.

HCC tissue studies provide complementary evidence from the immune-cell side. Siglec-9-associated signaling and functional inhibition have been demonstrated in tumor-infiltrating NK cells, while close spatial contact with Siglec-9 ligand-bearing cells was associated with adverse outcomes (17). Separately, Siglec-10hi TAMs were associated with CD8+ T-cell dysfunction, and Siglec-10-Fc treatment improved immune effector features and pembrolizumab-associated tumor-cell apoptosis ex vivo (18). These findings support investigation of glycan-directed interventions alongside PD-1/PD-L1 blockade. Their integration with sorafenib-associated glycan remodeling should be tested in matched tumor and immune-cell systems.

### 7.2 Postoperative molecular residual disease and early recurrence: a defined clinical state with an unresolved Siglec mechanism

Postoperative circulating tumor DNA (ctDNA) studies support the presence of a radiologically occult molecular residual disease (MRD) state after HCC resection (63–66). This state identifies patients at increased risk of recurrence, although it does not reveal the biological mechanisms that allow residual tumor cells to survive. In a cohort of 96 patients, recurrence occurred in 60.9% of patients with postoperative ctDNA positivity and in 27.8% of ctDNA-negative patients. Postoperative ctDNA positivity also independently predicted shorter disease-free survival (63). Hu et al. subsequently used a personalized, baseline-informed next-generation sequencing assay to examine perioperative plasma from 88 patients who underwent HCC resection (65). After a median follow-up of 80.7 months, postoperative MRD detected within 7 days after surgery was independently associated with recurrence (HR, 2.17; 95% confidence interval [CI], 1.09-4.30; P = 0.027) (65). A separate tumor-informed study included 125 patients. Among 34 evaluable patients who underwent resection, ctDNA was detected during the 2-12-week postoperative MRD window in 10 patients, all of whom subsequently developed recurrence (66). Detection during serial surveillance was also strongly associated with recurrence and preceded recurrence detection by AFP (66). A 2026 meta-analysis confirmed the overall prognostic value of postoperative ctDNA, while also showing substantial differences among studies in sampling time, assay design, and analytical sensitivity (64). These studies define a clinically important high-risk MRD state. The mechanisms supporting residual-cell survival and subsequent growth remain to be defined (**Table 3**, **Figure 3**).

**Table 3:** Postoperative ctDNA evidence for molecular residual disease in HCC.

Study	Design and population	Assay and window	Main result	Use in a combined MRD-glyco-immune framework	Ref
Ye et al., 2022	Resection cohort; HCC; n = 96 (BCLC 0/A/B, n = 75; BCLC C, n = 21)	Hybrid-capture NGS of tumor tissue, buffy coat and postoperative plasma; blood collected 7–10 days after surgery.	Recurrence: 60.9% ctDNA-positive vs 27.8% ctDNA-negative. DFS HR 6.074 (95% CI 2.648-13.929); OS HR 4.829 (1.508-15.466).	Use as an early residual-burden covariate or stratifier; it does not identify the immune mechanism sustaining residual cells.	(63)
Hu et al., 2025	Single-center resection cohort; n = 88; median follow-up 80.7 months	Baseline-informed personalized NGS; plasma before and within 7 days after surgery	Postoperative MRD was independently associated with recurrence: HR 2.17 (95% CI 1.09-4.30; P = 0.027), including clinically lower-risk groups.	Lock the early MRD time point and test whether tissue glyco-immune markers add information beyond it.	(65)
Abdelrahim et al., 2025	Retrospective real-world series across 11 centers; 125 patients and 721 plasma time points; 34 evaluable resection patients in the postoperative MRD analysis	Tumor-informed ctDNA; postoperative MRD at 2–12 weeks plus longitudinal surveillance	10/34 were MRD-positive; all 10 recurred. Serial ctDNA was strongly associated with recurrence and preceded AFP-based detection of recurrence.	Use longitudinal ctDNA to monitor residual disease alongside separate tissue glyco-immune measurements.	(66)
Buonopane et al., 2026	Systematic review and random-effects meta-analysis; 10 retrospective studies; n = 793 non-metastatic HCC patients treated with curative intent	Study-specific plasma assays and sampling times	Postoperative ctDNA positivity was associated with shorter RFS: pooled HR 4.48 (95% CI 2.56-7.82; I^2^ = 78%) and OS: HR 2.99 (1.94-4.61; I^2^ = 47%). Sensitivity 33-82%; specificity 41-100%.	Strong prognostic signal, but assay harmonization and prespecified timing are required for biomarker-stratified trials.	(64)

AFP, α-fetoprotein; BCLC, Barcelona Clinic Liver Cancer; CI, confidence interval; ctDNA, circulating tumor DNA; DFS, disease-free survival; HCC, hepatocellular carcinoma; HR, hazard ratio; I^2^, heterogeneity statistic; MRD, molecular residual disease; NGS, next-generation sequencing; OS, overall survival; RFS, recurrence-free survival.

Evidence from single-cell and spatial studies further suggests that recurrence depends on both residual tumor burden and the surrounding tissue environment (58, 67, 68). Early-relapse HCC contained KLRB1^+^ innate-like CD8^+^ T cells with weak cytotoxicity and limited clonal expansion. These cells differed from the more conventional exhausted CD8^+^ T-cell states found in primary tumors (67). Multi-omic spatial mapping also identified an oncofetal neighborhood centered on POSTN^+^ fibroblasts, together with nearby FOLR2^+^ macrophages and PLVAP^+^ endothelial cells (68). The corresponding tissue signature was associated with regulatory T-cell recruitment, early relapse, and differences in response to immunotherapy (68).

Additional evidence comes from residual HCC after TACE, which represents a different clinical setting from postoperative MRD. In this setting, PD-L1^+^ macrophages were spatially coupled to stem-like tumor cells, while macrophage-derived TGF-β1 promoted residual-cell persistence (58). Combined inhibition of PD-L1 and TGF-β eliminated residual disease in mouse HCC models (58). Although these studies did not assess sialoglycans or Siglec signaling, they show that residual HCC cells can be supported by spatially organized immune niches and that these niches may be therapeutically disrupted (58, 67, 68).

These observations support a testable model linking tumor-cell dissemination, subsequent growth, and immune escape. The association of early HCC recurrence with MVI is consistent with occult dissemination before resection (3). As discussed above, glycocalyx-dependent changes in cell contacts and matrix adhesion provide potential mechanisms affecting tissue escape and colonization. Experimental evidence from mammary tumor models further shows that a bulky glycocalyx promotes the growth of disseminated cells at distant sites (34). In HCC, studies of Siglec-9-associated NK-cell inhibition and Siglec-10hi macrophage states provide complementary evidence for impaired immune surveillance (17, 18). Therefore, we propose that, in a subset of HCCs, glycocalyx remodeling may facilitate dissemination and support growth at secondary sites, while compatible Siglec ligands and suppressive immune-cell states reduce clearance of surviving cells. This model connects the physical and immune consequences of the glycocalyx as potential contributors to residual-cell persistence and early recurrence.

Within this framework, postoperative ctDNA provides molecular evidence of residual disease and identifies increased recurrence risk, while tissue analysis can characterize candidate mechanisms of persistence (63). Prospective studies should combine longitudinal ctDNA sampling before and after resection with spatial profiling of glycans, their carriers, and immune-cell states in the resected tumor. Sampling should include MVI fronts and satellite nodules where available, with analysis of recurrent lesions to assess clonal relatedness and retention of the candidate phenotype. ST3GAL1 expression and Siglec-compatible ligand display should be evaluated alongside receptor-associated signaling and immune function, while adhesion and growth-related features could test the proposed physical component. The central clinical question is whether these features improve prediction of early recurrence beyond postoperative ctDNA, tumor size and number, vascular invasion, AFP, and other established pathological factors. Recurrence-free survival should be assessed prospectively, with the contribution of each component and their combined value evaluated separately.

### 7.3 Translational implications for adjuvant and perioperative therapy

Results from adjuvant immunotherapy trials highlight the need to select patients according to tumor biology as well as clinicopathological recurrence risk (69–71). At the prespecified interim analysis of IMbrave050, atezolizumab plus bevacizumab improved RFS compared with active surveillance in patients at high clinicopathological risk after HCC resection or ablation (HR, 0.72; adjusted 95% CI, 0.53-0.98) (69). However, this benefit was not sustained with longer follow-up (updated HR, 0.90; 95% CI, 0.72-1.12). OS remained immature, and the updated benefit-risk assessment did not support adjuvant use in an unselected high-risk population (70). In KEYNOTE-937, adjuvant pembrolizumab also did not improve RFS after resection or local ablation (median, 46.7 versus 45.5 months; HR, 1.06; 95% CI, 0.88-1.26; P = 0.719) (71). In contrast, the randomized phase 2/3 CARES-009 trial showed that perioperative camrelizumab plus rivoceranib improved event-free survival compared with surgery alone in patients with resectable HCC at intermediate or high risk of recurrence (median, 42.1 versus 19.4 months; HR, 0.59; 95% CI, 0.41-0.85) (72). The divergent findings across adjuvant and perioperative trials suggest that recurrence risk alone does not define a uniform treatment-responsive biological state, while treatment regimen, timing, and residual-disease context may also influence efficacy. Clinicopathological risk remains useful for identifying patients with a higher probability of recurrence, whereas biological markers may help identify the immune escape mechanisms that can be reversed by a specific treatment (**Table 4**).

**Table 4:** Adjuvant and perioperative immunotherapy trials in curative-intent HCC.

Trial	Population	Design and sample size	Regimen	Main efficacy result	Biomarker implication	Refs
IMbrave050	High recurrence risk after complete resection or ablation	Global randomized open-label phase 3; n = 668 (334 per group)	Adjuvant atezolizumab and bevacizumab every 3 weeks for 17 cycles vs active surveillance	Interim RFS HR 0.72 (adjusted 95% CI 0.53-0.98); updated HR 0.90 (95% CI 0.72-1.12). The interim RFS benefit was not sustained at the updated analysis.	The updated analysis did not support sustained RFS benefit in the selected high-risk population; predictive markers require treatment-by-biomarker interaction testing.	(69, 70)
KEYNOTE-937	Complete radiologic response after resection or local ablation; Child-Pugh A; ECOG 0-1	Global randomized double-blind phase 3; n = 959	Adjuvant pembrolizumab every 3 weeks for up to 17 cycles vs placebo	Median RFS 46.7 vs 45.5 months; HR 1.06 (95% CI 0.88-1.26; P = 0.719). The RFS endpoint was not met; OS was not formally tested under the multiplicity plan.	Adjuvant pembrolizumab did not improve RFS in the enrolled population; the trial did not test a sialylation-Siglec biomarker or strategy.	(71)
CARES-009	Resectable intermediate- or high-risk HCC (CNLC Ib-IIIa; BCLC A with tumors >5 cm, B, or selected C without main portal vein invasion or extrahepatic spread)	Randomized open-label phase 2/3 across 16 Chinese centers; n = 294 (148 vs 146)	Two neoadjuvant cycles of camrelizumab and rivoceranib, surgery, then adjuvant combination vs surgery alone	Median EFS 42.1 vs 19.4 months; HR 0.59 (95% CI 0.41-0.85; P = 0.004). OS was immature.	Differences in regimen, timing and disease context may contribute to the divergent efficacy observed across trials.	(72)

BCLC, Barcelona Clinic Liver Cancer; CI, confidence interval; CNLC, China Liver Cancer staging system; ECOG, Eastern Cooperative Oncology Group; EFS, event-free survival; HCC, hepatocellular carcinoma; HR, hazard ratio; OS, overall survival; RFS, recurrence-free survival.

Therefore, postoperative MRD offers a useful setting in which to test the contribution of the sialylation-Siglec axis to recurrence. Future prospective studies should combine postoperative ctDNA with tissue-based measurements of sialylated glycans, Siglec-expressing immune cells, and spatial receptor-ligand contact. This approach could determine whether a glyco-immune phenotype provides prognostic information beyond conventional pathology and residual tumor burden. It could also identify a biologically defined subgroup for future adjuvant studies (**Figure 3**).

## 8 Discussion and translational outlook

Moving the sialylation-Siglec axis toward clinical use in HCC requires a stepwise process. Four questions should be addressed in order: Can the glyco-immune phenotype be measured accurately and reproducibly? Does it predict recurrence beyond established risk factors? Does an intervention reach the intended target and produce the expected immune effect? Finally, does the biomarker identify patients who benefit more from a specific treatment (73, 74)? ST3GAL1, Siglec-7/9-compatible ligands, Siglec-9^+^ NK cells, and Siglec-10^hi^ TAMs are supported by current biological evidence, but none has been validated as an HCC biomarker or companion diagnostic (**Figure 4**) (17–19).

**Figure 4:**
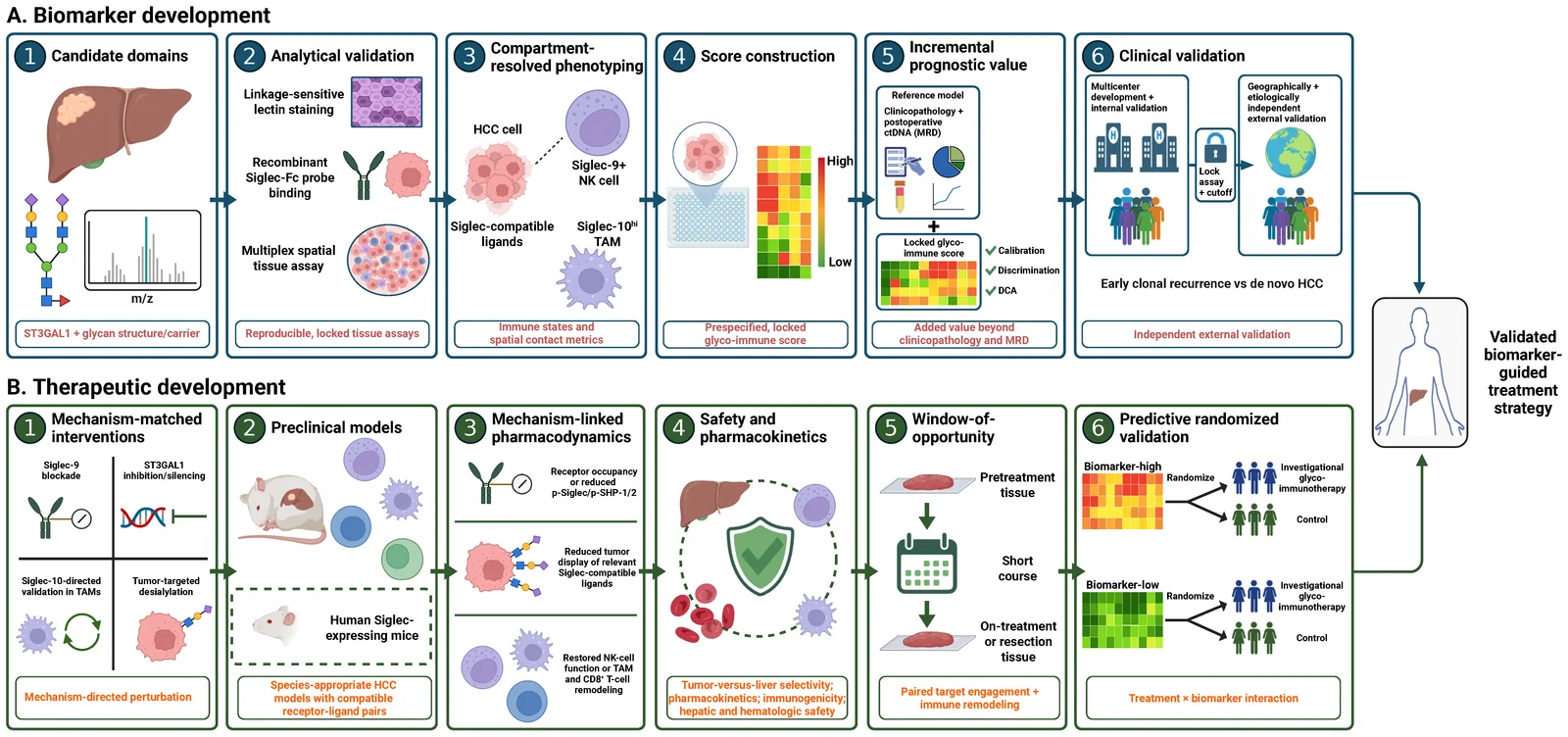
Translational roadmap for HCC sialylation-Siglec biomarkers and therapies. **(A)** presents a six-step biomarker development pathway. Candidate tumor and immune features are evaluated with reproducible tissue assays and combined into a compartment-resolved phenotype. These features are used to construct a prespecified glyco-immune score. The score is then tested for added prognostic value beyond clinicopathological factors and postoperative ctDNA, with assessment across etiologies and validation in independent patient populations. **(B)** presents a parallel six-step therapeutic development pathway. Mechanism-matched interventions are tested in models with compatible receptor-ligand pairs, including human tumor-immune-cell systems and appropriate mouse models, followed by pharmacodynamic assessment, safety and PK studies, paired tissue analysis in window-of-opportunity studies, and randomized evaluation of treatment-by-biomarker interactions. The two pathways aim to establish a validated biomarker-guided treatment strategy. ctDNA, circulating tumor DNA; DCA, decision curve analysis; Fc, fragment crystallizable region; HCC, hepatocellular carcinoma; MRD, molecular residual disease; m/z, mass-to-charge ratio; NK, natural killer; PK, pharmacokinetics; SHP-1/2, Src homology 2 domain-containing protein tyrosine phosphatases 1 and 2; Siglec, sialic acid-binding immunoglobulin-like lectin; ST3GAL1, ST3 β-galactoside α-2,3-sialyltransferase 1; TAM, tumor-associated macrophage; p-Siglec, phosphorylated Siglec; p-SHP-1/2, phosphorylated SHP-1 and SHP-2. Created in BioRender. Zhang, K. (2026) https://BioRender.com/en20usb.

### 8.1 Assay development: measuring tumor and immune features together

Assay development should first establish that the proposed markers can be measured reliably. In resection specimens, tumor-side assessment should combine ST3GAL1 expression with independent measurements of the glycocalyx. Linkage-sensitive lectin histochemistry can detect broad staining patterns associated with α2,3- and α2,6-linked sialylation (11, 27). Validated glycomic and glycoproteomic workflows can provide more detailed information on glycan composition and site-specific carriers (28). Lectin histochemistry can provide spatial evidence of broad sialylation patterns. Confident assignment of α2,3- and α2,6-linked sialic acids requires a linkage-specific method, such as differential derivatization followed by mass spectrometry (75). Recombinant Siglec-Fc probes can show whether cell-surface glycans are capable of binding a particular Siglec under defined assay conditions (19). However, in the HCC study, these probes did not resolve the complete glycan epitope or its glycoprotein or glycolipid carrier, and they did not directly show activation of endogenous Siglec receptors within tumor tissue (19). The immune-side assessment should measure Siglec-9^+^ NK cells and Siglec-10^hi^ TAMs together with their functional states (17, 18). Receptor abundance alone gives an incomplete view of inhibitory signaling. Relevant functional measurements may include NK-cell degranulation and cytokine production, macrophage suppressive markers, CD8^+^ T-cell activity, and phosphorylation of Siglec-associated signaling molecules (17, 18). Before a combined score or cutoff is fixed, studies should standardize tissue collection and processing, confirm antibody and lectin specificity, define signal normalization procedures, assess agreement between observers, control batch effects, and demonstrate reproducibility across laboratories.

Blood-based assays may allow repeated measurement during follow-up, but the biological source of circulating glycan signals must be established. Integrated plasma proteomics and N-glycoproteomics identified 32 differentially expressed intact glycopeptides in a discovery set of 11 patients with HCC and 11 healthy controls. In that small cohort, glycopeptide profiles distinguished HCC from healthy controls better than protein profiles (76). These findings support the feasibility of plasma glycoproteomic analysis, but they do not establish recurrence prediction, and circulating glycans may arise from the tumor, non-tumor liver, inflammatory cells, or other tissues (76). Candidate circulating sialoglycoproteins and extracellular-vesicle-associated Siglec ligands should therefore be compared with matched tumor tissue and tested against controls with cirrhosis or other chronic liver diseases. These assays should be evaluated alongside postoperative ctDNA. CtDNA provides information about MRD burden and has shown prognostic value after HCC resection in several independent cohorts (63, 65, 66). Glycan and Siglec measurements may provide a different type of information by describing the immune environment that supports residual-cell survival. Combining the two may help distinguish residual tumor burden from glyco-immune escape, but their added values must be demonstrated empirically.

### 8.2 Prognostic validation: predicting recurrence beyond pathology and MRD

Prognostic validation evaluates whether a biomarker provides information about recurrence risk within a defined clinical setting. Predictive validation evaluates whether treatment benefit differs according to biomarker status (73). Spatial analysis is central because Siglec signaling requires receptor-bearing immune cells to encounter appropriately presented sialoglycans. Single-cell and spatial studies in HCC have identified immune states associated with recurrence, oncofetal cellular neighborhoods, macrophage-tumor-cell niches in residual disease, and endothelial regions associated with MVI (58, 67, 68, 77). Within a multilayer biomarker framework, the PGAM2-sialylation-related signature described above provides a candidate transcriptional layer for future recurrence-focused validation, whereas defined glycan carriers and spatial ligand-receptor measurements can provide the mechanism-linked layer (29). Accordingly, a candidate tissue-based glyco-immune recurrence score could integrate tumor ST3GAL1 expression, structurally characterized or Siglec-binding-compatible ligands, the functional states of Siglec-9^+^ NK cells and Siglec-10^hi^ TAMs, and the frequency or distance of spatial contacts between ligand-bearing cells and Siglec-positive immune cells (17–19). Postoperative ctDNA should remain a separate measure of MRD and should be included with standard clinicopathological variables in the reference prognostic model (63, 65, 66). The main question is whether the glyco-immune score adds useful information beyond these existing measures.

Building on perioperative ctDNA studies, prospective validation should begin in a multicenter cohort undergoing curative-intent resection, with paired tumor and non-tumor liver tissue and longitudinal blood sampling (65). Plasma should be collected before surgery, within a prespecified postoperative window, and at fixed follow-up visits. Tissue and blood assays should be performed using standardized procedures and interpreted without knowledge of outcomes. RFS, defined as recurrence or death, should be the primary endpoint, with a prespecified analysis of early recurrence. For models incorporating postoperative ctDNA, follow-up should begin at a common landmark after the sampling window among patients alive and recurrence-free at that time. Later ctDNA measurements should be evaluated separately for updated risk prediction. Postoperative treatments should be recorded and addressed in prespecified analyses. Validation after ablation should be conducted separately, accounting for differences in tissue availability and local tumor progression.

The reference model should include tumor size and number, vascular invasion, AFP, differentiation, satellite lesions, resection margin status, liver function, and postoperative ctDNA status, with overlapping predictors handled during model specification. Candidate glyco-immune variables should then be added to determine their incremental value. Sample size should be planned according to the anticipated event rate, number of candidate parameters, and expected model performance (78). Model development should use penalization and bootstrap internal validation, with continuous measurements retained where possible. Added value should be assessed through calibration and discrimination at prespecified time points, together with decision-curve analysis at clinically relevant risk thresholds (79). Adhesion and growth-related measurements proposed above should initially be evaluated as exploratory additions. The final assay, model coefficients, and any decision thresholds should be fixed before external validation. Where recurrent tissue is available, comparison with the primary tumor should help distinguish clonal recurrence from a new primary tumor.

Etiology should be considered during biomarker development because HCC arises in different metabolic and immune environments. Studies of non-alcoholic steatohepatitis (NASH)-related HCC identified altered fatty acid and bile acid signaling, oxidative stress, and an immunosuppressive surrounding liver environment compared with other etiologies (80). Pfister et al. further demonstrated abnormal CD8^+^ T-cell activation and impaired tumor surveillance in NASH-associated HCC models treated with PD-1 blockade (81). A more direct link to sialylation was reported by Fan et al., who found an association between hepatitis C virus (HCV) infection and higher tumor ST3GAL1 expression (19). These findings provide a rationale for testing whether viral, alcohol-related, and metabolic HCC differ in sialyltransferase activity, glycan presentation, and the abundance and functional states of Siglec-positive immune cells. The resulting glyco-immune differences remain to be characterized directly across etiologies.

Validation cohorts should therefore include HBV-related, HCV-related, alcohol-related, and metabolic dysfunction-associated steatotic liver disease-associated HCC, with metabolic dysfunction-associated steatohepatitis documented where assessable. Coexisting causes should be recorded, together with viral suppression, antiviral treatment, alcohol exposure, metabolic comorbidities, fibrosis, and liver inflammatory activity. Prespecified analyses should compare biomarker distributions, spatial immune associations, and model calibration and discrimination across etiologies. Biomarker-by-etiology interactions should be evaluated when supported by adequate event numbers. External validation should include geographically distinct populations and sufficient representation of each major etiology, using the same assay and model before considering recalibration. Whether etiology modifies the relationship between glyco-immune status and checkpoint-inhibitor benefit should be examined separately within treatment-specific studies, as outlined in Section 8.4.

### 8.3 Therapeutic strategies, mechanistic validation, model selection, and safety

Therapeutic development should distinguish receptor blockade from interventions that alter ligand synthesis or remove surface sialic acids. Receptor-selective antibodies or small molecules offer a focused approach when suppression depends on a defined Siglec pathway. In HCC models, MTX-3937 reduced Siglec-9-SHP-1/2 signaling and restored NK-cell function, supporting further development of this strategy (17). Inhibiting ST3GAL1 instead modifies glycan biosynthesis: ST3GAL1 silencing reduced Siglec-7-Fc binding and improved NK-cell cytotoxicity and ADCC in sorafenib-resistant HCC models (19). These findings support a therapeutic contribution of ST3GAL1-dependent glycosylation, while the relevant molecular carriers and receptor dependence require further resolution. Soluble Siglec-10-Fc belongs to a different category because it sequesters ligands rather than selectively blocking endogenous Siglec-10 (18). Treatment development should therefore be guided by functional pathway dependence in the relevant immune compartment, alongside receptor and ligand expression.

Targeted enzymatic desialylation provides a broader ligand-directed strategy. Gray et al. demonstrated that human epidermal growth factor receptor 2 (HER2)-directed antibody-sialidase conjugates enhanced antitumor immunity *in vivo* (82). Stanczak et al. further showed that tumor desialylation repolarized TAMs and improved responses to immune checkpoint blockade in non-HCC models (83). These studies establish preclinical proof of concept for therapeutic glycocalyx editing. Unlike receptor-specific blockade, desialylation can reduce ligands available to several inhibitory Siglecs, potentially addressing overlapping suppressive pathways. Its activity, however, also extends to sialic-acid-dependent processes outside Siglec signaling. Translation to HCC should therefore establish a suitable tumor-targeting antigen, enzyme activity against the relevant tumor glycans, and preferential desialylation of tumor tissue over non-tumor liver. The glycan modifications discussed in Section 3, including O-acetylation, should be considered when measuring substrate removal and residual Siglec-binding activity.

Mechanistic validation should match the intervention. For receptor-directed agents, drug–receptor binding or occupancy should be linked to reduced inhibitory signaling and recovery of NK-cell or macrophage function. Receptor-selective blockade, genetic deletion, and receptor re-expression in primary human immune cells can test whether these effects depend on the proposed Siglec. For ST3GAL1-directed or enzymatic approaches, glycan changes, Siglec-Fc binding, and immune-cell function should be measured separately, with identification of the relevant glycoprotein or glycolipid carriers. Sialidase studies should include matched catalytically inactive constructs and controls for targeting-antibody and Fc effects. Because glycocalyx organization can regulate integrin signaling and adhesion (33), parallel measurements of tumor-cell adhesion, migration, and survival would help distinguish physical effects from Siglec-mediated immune effects. This distinction is particularly relevant to the dissemination and residual-cell survival model proposed in Section 7.2.

Model selection must account for species differences in Siglec biology. Human Siglec-7 and Siglec-9 lack simple one-to-one murine orthologues. Murine Siglec-E can reproduce selected inhibitory functions, but its expression patterns, ligand preferences, and biological activities do not fully match either human receptor; the Siglec-10–Siglec-G relationship is more clearly conserved (84). Immunocompetent orthotopic HCC models remain valuable for assessing integrated antitumor responses in a relevant liver environment. Human receptor-specific mechanisms should also be tested in primary human tumor–immune-cell cocultures and, where appropriate, humanized immune-system models or mice engineered to express human Siglecs. These systems require verification of receptor expression in the intended immune populations and compatibility with the tumor ligands being studied. Incorporating relevant chronic liver injury and etiological backgrounds would further connect preclinical testing to the heterogeneity discussed in Section 8.2. For recurrence-directed development, resection or ablation models should also be used to assess residual-cell persistence and subsequent recurrence.

Safety assessment should reflect the breadth and distribution of each intervention. Receptor-specific agents require evaluation of effects on normal Siglec-expressing immune cells, whereas glycan-directed approaches additionally require measurement of unintended glycan changes in non-tumor liver and circulating cells. Platelets deserve particular attention because desialylation can promote their hepatic clearance through the Ashwell receptor (85). HCC studies should therefore relate dose, tissue exposure, and duration of target engagement to hepatic function, blood counts, immune-cell trafficking, and tissue inflammation, especially in fibrotic or cirrhotic liver. Pharmacokinetics and immunogenicity are also important for repeated administration of enzyme-based platforms. Combination with PD-1/PD-L1 blockade or NK-cell-directed treatment should proceed from demonstrated complementary activity and acceptable combined toxicity. Together, these studies would provide the mechanistic and safety basis for the predictive clinical validation described in Section 8.4.

### 8.4 Predictive clinical validation: identifying patients who benefit from treatment

The contrasting results of IMbrave050, KEYNOTE-937, and CARES-009 place biomarker development in a predictive and treatment-specific setting (70–72). A high risk of recurrence can identify patients who may need additional therapy, while selection of the treatment regimen and treatment window requires further biological information. However, these trials do not demonstrate that a glyco-immune biomarker can identify treatment benefit. A clinically predictive biomarker must identify a difference in the relative treatment effect across biomarker groups. An association with recurrence or survival alone establishes prognostic value, not predictive value (73). Therefore, predictive validation should be performed within randomized perioperative or adjuvant trials using a finalized assay, a prespecified cut-off, and a formal biomarker-by-treatment interaction test. For the sialylation-Siglec system, such a trial should test whether a defined tissue or blood profile identifies benefit from a specific treatment strategy beyond standard clinicopathological risk and ctDNA-defined residual disease. Postoperative ctDNA positivity can identify patients with a high absolute risk of recurrence, but it does not show that their residual disease depends on the sialylation-Siglec pathway. MRD status and glyco-immune phenotype should therefore be treated as separate variables.

After assay reliability, prognostic value, target engagement, and hepatic safety have been established, an adjuvant trial could stratify patients according to both postoperative MRD status and glyco-immune score. Patients could then be randomized within each biomarker-defined stratum. This design would allow direct comparison of treatment effects between glyco-immune-high and glyco-immune-low disease. An enrichment trial limited to MRD-positive, glyco-immune-high patients could efficiently test whether a Siglec-directed strategy has activity in the proposed target population. However, because biomarker-low patients would be excluded, such a trial could not determine whether the glyco-immune score predicts a greater relative treatment effect. That question requires the inclusion of both biomarker-high and biomarker-low groups and formal interaction testing.

Early-phase trials should focus on safety, pharmacokinetics, tumor and immune-cell target engagement, immune remodeling, and ctDNA kinetics. Later-phase trials with adequate statistical power would be required to evaluate RFS and OS. This sequence would determine whether the sialylation-Siglec axis is clinically actionable in a defined subgroup of patients with HCC, while avoiding the assumption that it drives recurrence in all patients.

## 9 Conclusions

Current evidence supports a sialylation-Siglec immune-evasion network in HCC, with the strongest support for the Siglec-9-NK-cell axis and complementary evidence for Siglec-10^hi^ TAMs and ST3GAL1-dependent glycan remodeling. The physical effects of the glycocalyx on adhesion, migration, and survival provide a complementary basis for investigating dissemination and residual-cell persistence. However, direct clinical evidence linking this network to treatment resistance or postoperative recurrence remains limited. Future studies should identify the relevant glycan-carrier pairs, confirm receptor-specific signaling in species-appropriate HCC models, and prospectively integrate tissue glyco-immune markers with postoperative ctDNA and established clinicopathological factors across HCC etiologies before testing Siglec-directed strategies and tumor-targeted desialylation in biomarker-defined patient groups.

## Glossary

ADCC: Antibody-dependent cellular cytotoxicity
AFP: α-Fetoprotein
AGRN: Agrin
AKT: Protein kinase B
BCLC: Barcelona Clinic Liver Cancer
CI: Confidence interval
CNLC: China Liver Cancer staging system
ctDNA: Circulating tumor DNA
CTLA-4: Cytotoxic T-lymphocyte-associated protein 4
DAP12: DNAX activation protein 12
DCA: Decision curve analysis
DFS: Disease-free survival
ECOG: Eastern Cooperative Oncology Group
EFS: Event-free survival
EGFR: Epidermal growth factor receptor
Fc: Fragment crystallizable region
Gal: Galactose
GalNAc: N-Acetylgalactosamine
GlcNAc: N-Acetylglucosamine
HBV: Hepatitis B virus
HCC: Hepatocellular carcinoma
HCV: Hepatitis C virus
HER2: Human epidermal growth factor receptor 2
HR: Hazard ratio
IFN-γ: Interferon-γ
IL: Interleukin
iPSC: Induced pluripotent stem cell
ITIM: Immunoreceptor tyrosine-based inhibitory motif
LacNAc: N-Acetyllactosamine
LAG-3: Lymphocyte activation gene 3
MCAM: Melanoma cell adhesion molecule
miR-26a: MicroRNA-26a
MMPs: Matrix metalloproteinases
MRD: Molecular residual disease
mTOR: Mechanistic target of rapamycin
MVI: Microvascular invasion
NASH: Non-alcoholic steatohepatitis
Neu5Ac: N-Acetylneuraminic acid
NGS: Next-generation sequencing
NK: Natural killer
OS: Overall survival
PD-1: Programmed cell death protein 1
PD-L1: Programmed death-ligand 1
PGAM2: Phosphoglycerate mutase 2
PK: Pharmacokinetics
PNA: Peanut agglutinin
RFS: Recurrence-free survival
SHP-1/2: Src homology 2 domain-containing protein tyrosine phosphatases 1 and 2
Siglec: Sialic acid-binding immunoglobulin-like lectin
SR: Sorafenib-resistant
ST3GAL1: ST3 β-galactoside α-2,3-sialyltransferase 1
ST3GAL6: ST3 β-galactoside α-2,3-sialyltransferase 6
ST6GAL1: ST6 β-galactoside α-2,6-sialyltransferase 1
ST6GALNAC4: ST6 N-acetylgalactosaminide α-2,6-sialyltransferase 4
STRIDE: Single Tremelimumab Regular Interval Durvalumab
SYK: Spleen-associated tyrosine kinase
TACE: Transarterial chemoembolization
TAM: Tumor-associated macrophage
TGF-β: Transforming growth factor β
TGFBR2: Transforming growth factor β receptor 2
TIGIT: T-cell immunoreceptor with Ig and ITIM domains
TIM-3: T-cell immunoglobulin and mucin domain-containing protein 3
TNF-α: Tumor necrosis factor α
Treg: Regulatory T cell.
